# A Comprehensive Review of Fortification, Bioavailability, and Health Benefits of Folate

**DOI:** 10.3390/ijms26167703

**Published:** 2025-08-09

**Authors:** Jiarun Li, Hanying Duan, Hosahalli Ramaswamy, Chao Wang

**Affiliations:** 1 Department of Food Science and Technology, Jinan University, Guangzhou 510632, China; ppr945@stu2022.jnu.edu.cn (J.L.); tduhy@jnu.edu.cn (H.D.); 2 Department of Food Science and Agricultural Chemistry, Macdonald Campus of McGill University, Montréal, QC H9X 3V9, Canada; hosahalli.ramaswamy@mcgill.ca

**Keywords:** folate, bioaccessibility, folate enrichment, health benefits, homocysteine, antioxidant, anti-inflammatory

## Abstract

Folate is an essential vitamin involved in one-carbon metabolism. It can be acquired from many food sources or in synthetic form. A wide range of processing methods have been studied to improve the bioaccessibility and bioavailability of folate in foods, yet this is often accompanied by a decrease in stability. Encapsulation technology has emerged as an effective solution for protecting folate from degradation and liberation while also improving its bioavailability. Folate deficiency is a prevalent phenomenon worldwide, particularly in underprivileged countries, leading to various health problems, such as neural tube defects. Thus, folate was fortified through both exogenous addition and biofortification. Gene editing technology, especially CRISPR-Cas9, has great promise in this field when compared to transgenic engineering, because transgenic engineering may pose safety concerns and environmental risks. While ongoing research has identified additional potential effects of folate, the dosage and duration remain important factors to consider for optimal health outcomes. The mechanisms of how folate promotes the production of neurotransmitters associated with the gut microbiota–brain axis and reduces depression are not well understood. In addition to folate alone, there may be synergistic effects of combined supplementation of folate and other nutrients or medications, but this is not yet fully clarified and requires further examination. This review summarizes the food sources, enrichment, bioaccessibility, and bioavailability of folate. Furthermore, the health benefits of folate, including neural tube protection, cardiovascular protection, neuroprotection, anti-cancer, immune response augmentation, and gut homeostasis maintenance, with their potential bioactivity mechanisms, are discussed.

## 1. Introduction

Folate, also known as vitamin B_9_, is a water-soluble vitamin that is crucial for the development of organisms. It acts as a coenzyme involved in the one-carbon transfer reactions for the synthesis and repairment of nucleotides, the methylation of amino acids, proteins, and lipids, as well as the formation of red blood cells (RBCs) [1].

At present, folate deficiency is a widespread problem among women of reproductive age globally, and it is particularly significant in some developing countries, such as Ethiopia and Kenya, leading to a variety of health issues [2,3]. Therefore, the U.S. Food and Drug Administration (FDA) recommends a daily intake of 400 μg of dietary folate equivalents (DFE) for adults and 600 μg for women of reproductive age to prevent these health complications and ensure the proper functioning of the body [4]. Apart from treating neural tube defects (NTDs), both in vitro and in vivo studies found that in addition to homocysteine (Hcy) levels, folate levels were also linked to oxidative stress and inflammation [5,6,7]. Consequently, with the growing interest in folate, its health benefits have been investigated extensively, and the research findings have demonstrated that folate can be used to treat anemia, dementia [8], cardiovascular diseases (CVDs) [9], and certain types of cancer [10,11], along with maintaining gut homeostasis [12] and enhancing the immune response [13].

Although the terms “folate” and “folic acid” (FA) are usually used interchangeably, they are not exactly the same thing. Folate refers to naturally occurring forms of tetrahydrofolate (THF) in foods, while FA is the fully oxidized form of folate commonly used in dietary supplements and fortified foods with higher stability, bioaccessibility, and bioavailability [14,15]. However, there is growing evidence suggesting that this synthetic FA could mask the symptoms of vitamin B12 deficiency in a minority of patients due to its difficulty being metabolized by individuals with certain genetic variations [16]. 5-Methyl-tetrahydrofolate (5-MTHF) is the predominant form of folate in most plants since it can be used by the body for various biochemical processes, while its bioaccessibility was not as high as that of other folate vitamers [17,18]. Therefore, it is necessary to employ all kinds of handling methods to make it bioaccessible, with fermentation, deglutamylation, and thermal treatment being the most popular methods used nowadays. Encapsulation technology, a technique that involves trapping folate within a protective coating, has been shown to improve the bioavailability of folate, as it can protect folate from gastric juice and deliver folate to the target site of absorption in the body in a controlled manner. In addition, encapsulation reduces the degradation and liberation of folate by heat and/or UV radiation during processing and storage [19]. Increasing the folate content of foods is also a viable approach; in particular, gene editing technologies, such as CRISPR-Cas9, can precisely modify plant genomes to facilitate folate biosynthesis. In addition, gene editing technologies are generally considered safer and more efficient than transgenic engineering, and they have not encountered excessive regulatory hurdles to date [20].

Herein, this review discusses the structure, dietary sources, fortification, bioaccessibility, and bioavailability of folate, with special attention paid to summarizing the most recent research on its health benefits, aiming to provide a comprehensive understanding of the importance of folate in human health and nutrition.

## 2. Structure and Dietary Sources of Folate

**Figure 1:**
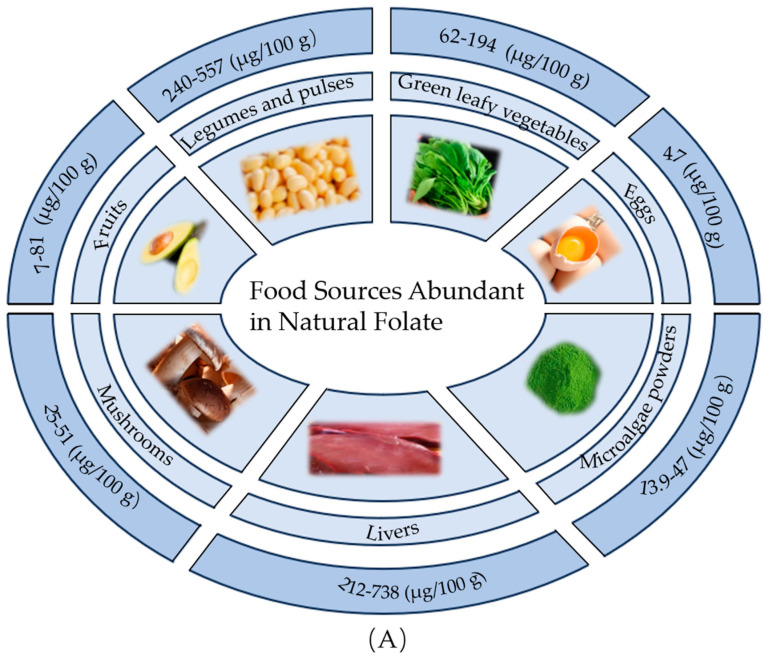

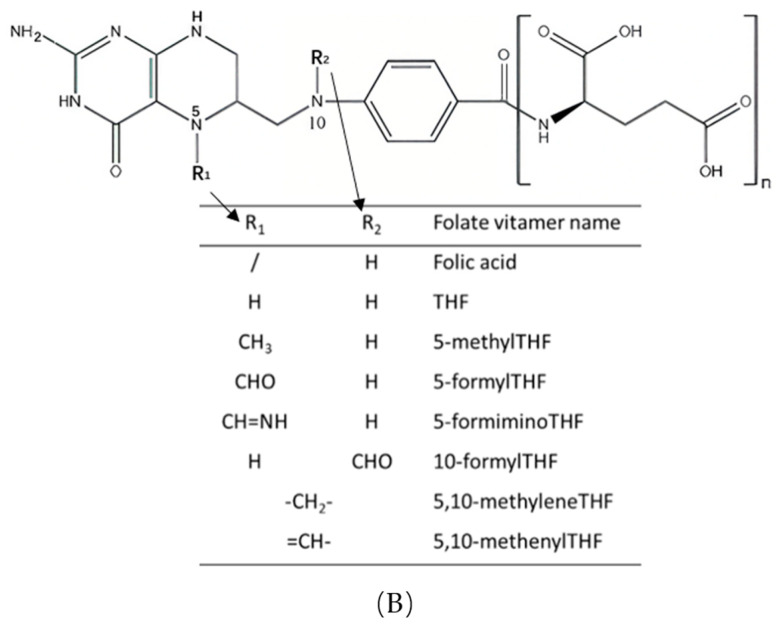
(**A**) Food sources abundant in natural folate. (**B**) The chemical structure of folates. R1 and R2 denote two functional groups of folates. The specific functional groups attached to the folate molecule define its folate vitamer names.

Chemically, folate is composed of a *p*-aminobenzoate (*p*ABA) molecule linked to a pteridine ring and a polyglutamyl chain (a variable number of glutamate moieties) (Figure 1). The structure of folate varies with the oxidation or reduction state at the N5 and/or N10 positions of the pteridine ring as well as the length of the polyglutamyl chain. These variations in structure impact the biological activity of folate, with the reduced forms being more biologically active than the oxidized forms.

The main natural dietary sources of folate are green leafy vegetables, fruits, legumes and pulses, livers, and eggs. According to the data provided by the USDA [4] shown in Table S1, livers are the most abundant sources of folate among the common foods listed (212–738 mg/100 g), followed by legumes and pulses (240–557 mg/100 g), and green leafy vegetables (62–194 mg/100 g). Specifically, raw goose and duck liver have the highest folate content, at 738 mg/100 g. In contrast, the amount of folate found in fruits and eggs is comparatively lower, ranging from only 7 to 81 mg/100 g. Mushrooms (25–51 μg/100 g) and microalgae (13.9–47 μg/g) represent valuable emerging folate sources [21,22]. Adding them to the diet significantly increases several micronutrients (B vitamins, selenium, copper, potassium, and fiber) [23,24], without impacting energy, sodium, or fat intake [25]. Beyond these nutritional advantages, both mushrooms and microalgae offer significant environmental benefits due to their sustainable, cyclical cultivation methods and serve as versatile raw materials for food processing [26]. These qualities align well with growing trends in plant-based nutrition and eco-friendly food systems [27]. In addition to food folate, synthetic FA is added to grains and cereals in certain countries to meet the mandatory fortification requirement and to prevent folate deficiencies in the general population. Folate can also be synthesized in vivo through the action of gut bacteria in the large intestine by converting *p*ABA to dihydrofolate (DHF), THF, and finally 5-MTHF and other derivatives [17]. However, the amount produced is typically not enough to meet daily requirements, so it is important to consume a variety of folate-rich foods and, if necessary, consider supplementation to ensure an adequate intake for overall health.

## 3. Folate Fortification

Folate fortification is often achieved by incorporating synthetic FA into staple foods such as rice, wheat, maize, and sorghum prior to milling or processing. This practice is cost-effective since it requires minimal changes to existing food production processes and infrastructure and has been successful in reducing the incidence of NTDs in populations where it has been implemented, including the US, South Africa, and Canada [28,29,30].

Folate biofortification, as opposed to exogenous addition, is a method where crops are selectively bred or genetically modified to increase their natural folate content. Germination, or sprouting, is a traditional method used to increase the folate content of plants. Briefly, germination speeds up the production of folate in plant cells by activating endogenous enzymes, stimulating metabolic pathways, and reducing anti-nutritional factors. A recent study showed that the total folate content of quinoa from the QL-2 variety increased by 9.07 times to reach 1735.6 mg/100 g dry weight (DW) on the sixth day of germination, compared to the folate content of its seed [31]. Germination has also observed an increase in total folate content in other plants, such as brown lentils, white beans, black-eyed peas, wheat, and rye [32,33].

The folate content of various foods can also be enhanced through a process involving folate-producing bacteria. These bacteria can thrive in the fermentation environment and initiate de novo folate synthesis, which typically consists of four main metabolic pathways [34]. Lactic acid bacteria (LAB) are one of the most common types of bacteria involved in folate synthesis, known for their higher efficiency and safety compared to other bacteria. Moreover, LAB contributes to improved flavor, texture, preservation, and other nutritional values of food [35]. Mosso, et al. [36] developed a vegan product made with Andean potato, amaranth, and chia and fermented with *Lactobacillus sakei* CRL2210. The results showed that after 24 h of fermentation, the folate content amounted to 209.8 mg/100 g, which corresponds to 52% of the recommended dietary allowance (RDA) for an adult consuming a 100 g serving. It was also found that the addition of *Saccharomyces cerevisiae* in making tef injera, a traditional Ethiopian fermented staple food, significantly increased the folate content by over 3-fold after four days of fermentation, reaching 169 mg/100 g DW, with enhanced overall sensory acceptance [37]. Likewise, Hjortmo, et al. [38] showed that the folate content of white wheat bread fermented with a strain of *Saccharomyces cerevisiae* CBS7764, which was cultured in specialized medium and harvested during the respiratory fermentation phase prior to dough preparation, was three to five times higher compared to bread fermented with commercial baker’s yeast. The fermentation of date palm wine with yeast resulted in an increase in the amount of 5-MTHF from 0 to 0.32 mg/L on the fifth day [39]. Simultaneously, the content of 5-formylTHF (5-FTHF) consistently dropped by roughly 100 mg/L due to degradation caused by newly produced enzymes or compounds such as oxidases and formyl transferases.

Folate enrichment in plants through genetic biofortification has gained much attention since it offers a sustainable solution to address folate deficiency in populations that rely heavily on staple crops. Genetic biofortification can be classified into three categories, namely, conventional breeding, metabolic engineering, and gene editing [40]. Conventional breeding involves selecting and crossing plants that possess desirable traits to produce offspring with an improved folate content or a more favorable ratio of folate vitamers. Quantitative trait loci (QTLs), in this case, serve as a valuable tool for identifying regions of the genome associated with the folate content and can be used to develop markers for marker-assisted selection (MAS) in breeding programs; on the other hand, by using the molecular markers that are closely associated with these QTLs, breeders can efficiently screen large populations for the presence of desired traits for the future generations of plants [41]. This approach is successful in crops with a significant amount of genetic diversity, such as maize, rice, and soybeans [42,43,44]. For instance, in a segregated population of two maize lines (one with a high folate content and the other with a low folate content) crossed, two folate QTLs were obtained from chromosome 5, and they contributed 41.6% of the phenotypic variance in 5-FTHF [44]. Nevertheless, the process can be time-consuming and labor-intensive, as it requires multiple generations of breeding to achieve the desired outcomes.

Metabolic engineering involves the introduction of specific genes into plants to optimize the folate biosynthesis pathways for desired outcomes. GCHI, ADCS, HPPK/DHPS, and FPGS are key enzymes involved in the process and are commonly targeted for manipulation to increase the folate content [45]. Dong, et al. [46] observed that overexpressing GCHI or FPGS alone increased the folate content in rice by 6.1-fold and 1.5-fold, respectively. Another study showed that overexpression of GCHI alone led to a 2-fold increase in the total folate content in potatoes, while overexpression of ADCS alone did not result in a significant increase. Interestingly, simultaneous overexpression of GTPCHI and ADCS ultimately resulted in a 3-fold increase in the total folate content [47]. Therefore, it is crucial to manipulate multiple genes in different pathways to replete the biosynthetic intermediates and promote folate accumulation.

In recent years, gene editing technology has experienced notable progress and increased interest due to the development of clustered regularly interspaced short palindromic repeats and CRISPR-associated protein 9 (CRISPR-Cas9). This technique enables precise modifications to the DNA sequence (e.g., gene knock-out or knock-in) within the genome of an organism and reduces the occurrence of undesired outcomes such as the “off-target effect” in metabolic engineering [48]. In addition, as a transgene-free technique, gene editing could potentially alleviate concerns related to genetically modified organisms (GMOs) and provide a more acceptable approach, and regulatory authorities might adopt a more lenient attitude towards its use in the food industry. At present, CRISPR-Cas9 had successfully caused mutations in rice, tomatoes, and potatoes; notably, some gene mutations were passed to the next generation without any detectable new mutation or reversion [49,50]. Nevertheless, there have been few investigations into the use of CRISPR-Cas9 in folate biofortification, so further research is required to assess the potential benefits and risks associated with this application.

## 4. Bioaccessibility and Bioavailability of Folate

Pharmacokinetics defines bioaccessibility as the proportion of a substance that is released from the food matrix and can be absorbed by the small intestine; on the other hand, bioavailability refers to the proportion of an ingested substance that enters the systemic circulation and is available for metabolism and storage [51]. The determination of folate bioaccessibility is generally achieved through in vitro models, which consist of static and dynamic digestion models, both of which aim to simulate the conditions of the GI tract. Generally, static digestion models like INFOGEST consist of distinct phases, namely oral, gastric, and intestinal digestion, each characterized by specific pH and enzymes. Dynamic models, such as the TNO gastro-intestinal model (TIM-1), not only incorporate the physiological conditions that exist in static models but also mimic other digestive processes such as mixing and peristalsis, making them a more accurate model for studying the bioaccessibility of folate as well as the stability of folate vitamers. In contrast, animal models (mainly pigs) as well as human interventions are used to assess the bioavailability of folate since they are capable of investigating the pharmacokinetics of folate and establishing dose–response relationships.

**Figure 2:**
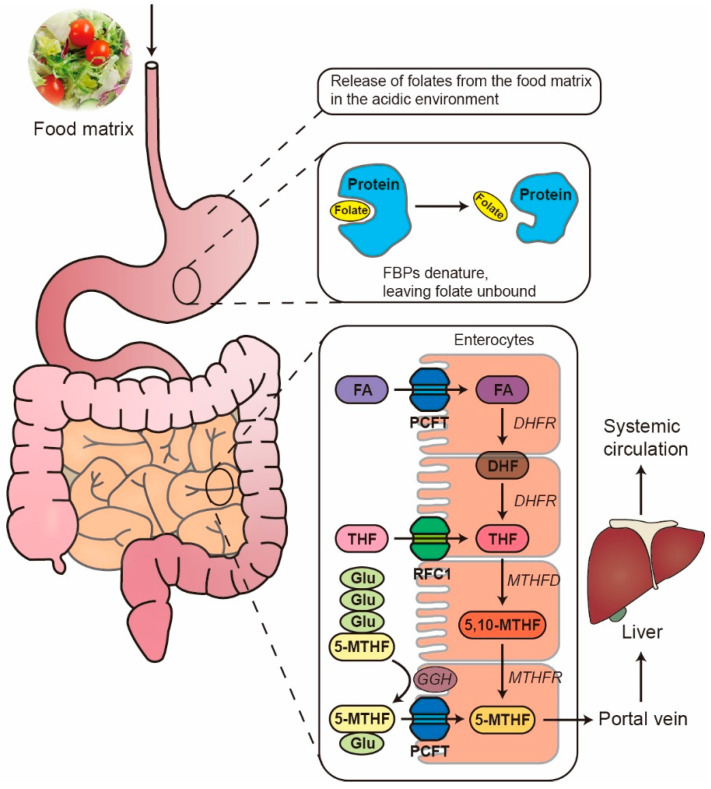
The process of digestion and absorption of folate in the gastrointestinal tract. Once the food matrix enters the stomach via the esophagus, folates are liberated from the food matrix in the presence of hydrochloric acid. For example, some folate-binding proteins (FBPs) are denatured during gastric digestion, allowing the release of bound folates. In the small intestine, FA and tetrahydrofolate (THF) are transported across the cell membranes of enterocytes via two types of folate transporters: the proton-coupled folate transporter (PCFT) and the reduced folate carrier (RFC1). Polyglutamates are deconjugated into monoglutamates with the help of γ-glutamylhydrolase (GGH), located on the brush border of the enterocytes. All absorbed folate vitamers are converted to 5-MTHF in the enterocytes and exported to the liver via the portal vein. The folates are then involved in the systemic circulation and delivered to various cells, where they participate in different physiological pathways. Other abbreviations: DHF, dihydrofolate; DHFR, dihydrofolate reductase; Glu, glutamate; MTHFD, methylenetetrahydrofolate dehydrogenase; MTHFR, methylenetetrahydrofolate reductase; 5,10-MTHF, 5,10-methylenetetrahydrofolate.

Figure 2 illustrates the mechanism of the digestion and absorption of folate in the gastrointestinal tract. The bioaccessibility and bioavailability of folate are influenced by two kinds of factors: exogenous and endogenous factors. Exogenous factors involve the food matrix, nutrient interaction, processing method, glutamyl chain length, and the form of folate.

Presently, a number of studies are focusing on exogenous factors since their improvement can greatly increase the bioaccessibility and bioavailability of folate. Exogenous factors involve the food matrix, nutrient interaction, processing method, glutamyl chain length, and the form of folate; endogenous factors involve the pH of the GI tract, digestive enzyme activity, and genetic factors such as MTHFR C677T [52,53]. The MTHFR C677T gene variation can affect the activity of MTHFR, thereby influencing folate metabolism. The enzyme activity of the CC genotype (wild type) is normal, and the folate metabolic capacity is also normal. The enzymatic activity of the CT genotype (heterozygous mutation) dropped to approximately 65%, and the folate utilization capacity moderately declined. The enzymatic activity of the TT genotype (homozygous mutation) is only 30%, and the folate metabolic capacity is severely impaired [54]. Studies have shown that the blood folate level of individuals with the TT genotype is approximately 16% lower than that of individuals with the CC genotype [55]. The average homocysteine level of individuals with the TT genotype of the MTHFR C677T gene was 19.4 µmol/L, while that of individuals with the CC genotype was 13.9 µmol/L [56]. This difference is mainly due to the reduced MTHFR enzyme activity in individuals with the TT genotype, which leads to a decrease in the production of 5-MTHF and subsequently affects the remethylation of homocysteine. Individuals with the TT genotype may face a higher risk of cardiovascular diseases [57].

In terms of the impact of different folate forms, the bioaccessibility of FA and formyl folates is higher than that of methyl folates, and they must undergo conversion into the bioactive form 5-MTHF before being utilized by the body. Due to genetic variation, the effectiveness of this conversion process varies among individuals, and therefore, it might be beneficial to supplement preformed 5-MTHF for better utilization. Nevertheless, 5-MTHF, one of the predominant forms of folate found in many foods, is susceptible to degradation and/or oxidation throughout the digestive process, resulting in reduced overall folate bioaccessibility [58]. In further investigation, the researchers found that the spiking of gallic acid in white wheat bread during in vitro digestion effectively enhanced the stability of 5-MTHF, resulting in a recovery up to 85% [18]. The addition of other reducing agents, such as ascorbic acid, was also reported to be effective in improving the bioaccessibility of folate in spinach and camembert [59].

Polyglutamates have a strong tendency to bind with proteins, forming folate-binding proteins (FBPs). Despite the fact that FBPs prevent the degradation of FA in the GI tract and thus improve stability, they hinder folate from deconjugation by γ-glutamylhydrolase (GGH) located in the brush border of the small intestine, thus affecting folate bioaccessibility and bioavailability. Arkbage, et al. [60] reported that the bioaccessibility was 82% in yoghurt fortified with FA and 5-MTHF using the TIM-1 model. However, after the addition of FBPs, the bioaccessibility decreased to 34% and 57% for FA-fortified and 5-MTHF-fortified yoghurt, respectively, indicating the negative effect of FBPs in folate-fortified yoghurt. de Jong, et al. [61] reported that dietary interventions in populations with FA-fortified UHT milk (FBPs denatured) and pasteurized milk (FBPs retained) increased the serum folate level (by 7.0 nM and 6.6 nM) and the RBC folate level (by 32 nM and 36 nM). Additionally, these interventions resulted in a reduction in the plasma Hcy levels by 0.88 mM and 0.89 mM, respectively, suggesting that FBPs in FA-fortified milk did not affect the bioavailability. Collectively, these results implied that FBPs may have different effects on the bioaccessibility and bioavailability of dairy products, which could be due to the differences in the digestion models used and the composition of dairy products.

Typically, folate present in food is bound to other components such as carbohydrates, proteins, or lipids, creating a matrix that traps the folate. Therefore, it is crucial to alter the structure of the matrix through various processing methods to liberate folate and enhance its bioaccessibility. Liu, et al. [62] reported that the bioaccessibility of folate in faba bean, oat, rye, and wheat increased from 42–67% to 78–123% after a 10 min heat treatment in close tubes. This increase can be mainly attributed to the release of oxidized folate vitamers from the food matrix caused by the heat treatment. Notably, heat treatment did not destroy 5-MTHF in faba bean, which explained its high bioaccessibility (123%), and therefore faba bean can be considered an important dietary source of folate. Fermentation is another promising method to improve both the folate content and bioaccessibility. While tofu had a relatively high folate bioaccessibility of 82%, the folate bioaccessibility of tempeh, which was soaked in LAB and fermented with pure fungi for 48 h, was much higher, reaching 103% [63]. Food processing can improve folate bioaccessibility by making the food structure loose and porous, but this improvement comes at the cost of reduced stability of folate. In the case of West African cereal-based fermented foods, those with a solid or semi-solid structure usually had higher folate bioaccessibility, presumably due to their ability to tolerate the low pH in the stomach and finally reach the small intestine where they can be absorbed; in contrast, folate in foods like porridges was liable to be degraded during the gastric phase [64]. Hence, it is vital to achieve a balance between bioaccessibility and stability.

The natural form of 5-MTHF found in most fruits and vegetables are polyglutamates. However, polyglutamates are less bioavailable than mono- and di-glutamates, so deglutamylation is necessary to improve their bioavailability. Wang, et al. [65] treated vegetables with 5-MTHF initially in the form of 4–6 units of polyglutamates with high hydrostatic pressure (HHP) and observed that the mono- and di-glutamates of carrots increased by 23-fold and 32-fold, respectively, at 600 MPa/5 min. Deglutamylation was also found in the cauliflower and carrot greens. In addition, the researchers found that juicing vegetables can also induce deglutamylation of 5-MTHF, especially in turnips and turnip greens. This led to a significant increase in the proportion of mono- and tri-glutamate, without causing a substantial decrease in the total folate content [66]. Deglutamylation can also take place in broccoli during blanching at a low temperature for a long time (LTLT) in an acidic condition. This may be linked to the maintenance of GGH activity at a low pH and temperature [67]. A more recent study found that exogenous ethylene can stimulate the conversion of 5-MTHF polyglutamates to monoglutamates in ripe fruit, along with the production of endogenous ethylene [68]. Nevertheless, McKillop, et al. [69] suggested that deglutamylation was not a limiting factor in the bioavailability of food folates. In their randomized crossover trial, no significant difference in the plasma folate response was observed among individuals consuming foods with different ratios of monoglutamate to polyglutamate. It is worth noting that this trial included only 13 male subjects; therefore, future studies should validate the effect of deglutamylation on folate bioavailability in a larger and more diverse population.

In recent years, with the advancement of encapsulation technology, researchers have developed a variety of micron- to nanoscale FA delivery systems, including particles [70], capsules [71], hydrogels [72], and electrospun fibers [19]. These systems involve the use of biocompatible materials that can protect FA from degradation in the GI tract and facilitate its controlled release in the body, leading to improved bioavailability. A dispersed FA system designed by Osojnik Crnivec, Istenic, Skrt, and Poklar Ulrih [72] achieved an encapsulation efficiency of up to 100% (encapsulated in liposomes). In addition, the researchers discovered that the dispersed FA encapsulated in alginate-pectin microbeads was pH-dependent, i.e., the FA was prevented from solubilization in the stomach but was fully released in the intestine due to the swelling of alginate gels. Another study suggested that the proportion of alginate and pectin encapsulating FA also affects the pharmacokinetic properties, or the bioavailability, with 100% alginate and 50% alginate−50% pectin nanoparticles being suitable for delivery of FA to the duodenum for absorption via the proton-coupled folate transporter (PCFT), and 100% alginate being suitable for the delivery to the terminal ileum via the reduced folate carrier (RFC1) (Figure 2) [70]. The encapsulation of FA into carboxymethylcellulose (CMC)/polyethylene oxide (PEO) nanofibers using electrospinning improved the thermal stability, as demonstrated by a reduction in the melting temperature and enthalpy of the PEO and an increase in the reaction heat of CMC. The release of FA under acidic environments was significantly decreased, with 6.16% at pH 3.0 and 13.56% at pH 5.6 after 30 days of exposure [19]. Furthermore, the encapsulation system was able to protect FA against UV radiation (21% loss of encapsulated FA vs. 100% loss of free FA after 60 min of exposure). Overall, delivery of FA via encapsulation can achieve higher bioavailability, photostability, thermal stability, and pharmacokinetic properties compared to conventional FA delivery approaches and is therefore a promising method for preventing loss of FA during processing and storage, as well as for delivering FA to the target site of absorption.

## 5. Health Benefits of Folate

**Figure 3:**
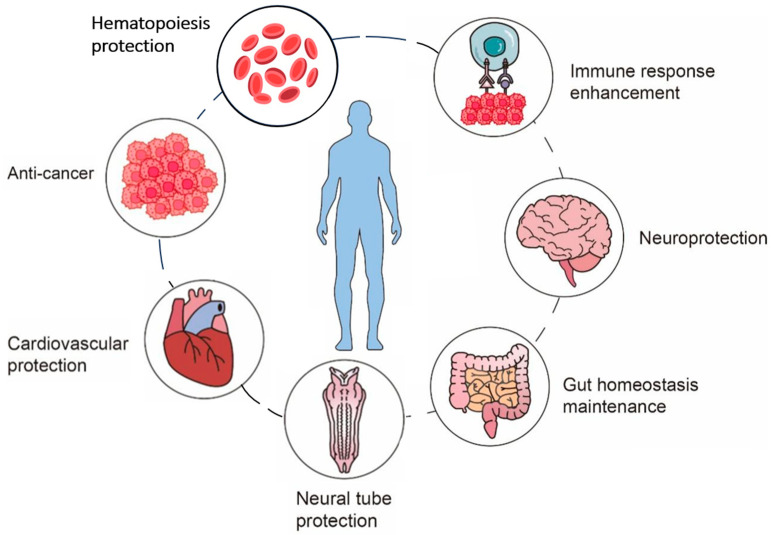
Potential health benefits of folate.

To date, folate has been found to show positive effects in cardiovascular, hematopoiesis, neurological, and neural tube protection, as well as in anti-cancer, gut homeostasis, and immune response, which will be discussed in detail below (Figure 3).

### 5.1. Cardiovascular Protection

**Figure 4:**
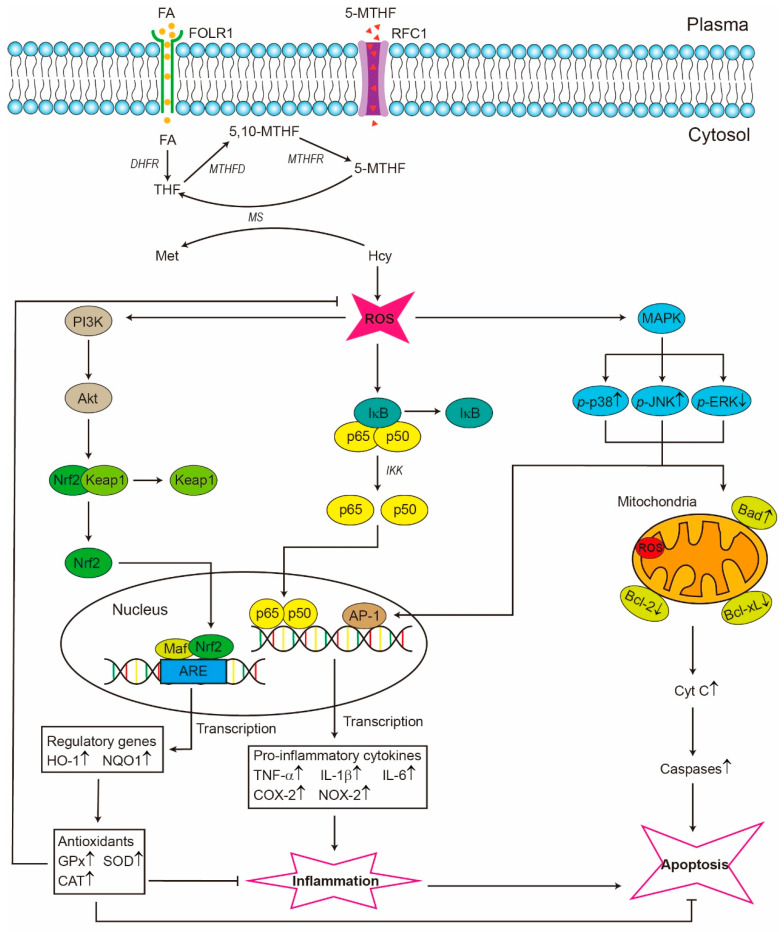
Summary of the mechanism of folate on homocysteine (Hcy) levels, oxidative stress, inflammation, and apoptosis. Cellular uptake of 5-MTHF occurs via the folate receptor (FOLR) and the reduced folate carrier (RFC) present on the cell membrane. In the cytoplasm, 5-MTHF donates its methyl group to Hcy, forming THF and methionine (Met). Accumulation of Hcy can promote the formation of reactive oxygen species (ROS), leading to oxidative stress. ROS can activate three signaling pathways in cells: ① Phosphoinositide 3-kinase/protein kinase B/nuclear factor erythroid 2-related factor 2 (PI3K/Akt/Nrf2) pathway: ROS activates PI3K and Akt. Upon activation by Akt-mediated phosphorylation and/or Keap1 oxidation by ROS, Nrf2 is released from Kelch-like ECH-associated protein 1 (Keap1) and translocated into the nucleus, where it binds to antioxidant response elements (AREs) in the promoter regions of its target genes. AREs thereby initiate the transcription of cytoprotective enzymes such as NAD(P)H quinone oxidoreductase 1 (NQO1) and heme oxygenase-1 (HO-1), thereby enhancing the production of antioxidants. These antioxidants are able to mitigate inflammation apoptosis and the generation of ROS. ② Nuclear factor-kappa B (NF-κB) pathway: the IκB kinase (IKK) complex was activated by ROS, which releases NF-κB dimers (p50 and RelA/p65) from their inhibitory IκB proteins. p65 and p50 are then translocated into the nucleus, where they bind to specific DNA sequences called κB sites, thereby regulating the transcription of a range of pro-inflammatory cytokines, ultimately leading to inflammation and promoting apoptosis. ③ Mitogen-activated protein kinase (MAPK) pathway: the phosphorylation of p38, c-Jun N-terminal kinase (JNK), and extracellular signal-regulated kinase (ERK) is up-regulated. It results in the inactivation of anti-apoptotic proteins B-cell lymphoma 2 (Bcl-2) and B-cell lymphoma-extra large (Bcl-xL), as well as the activation of pro-apoptotic proteins such as Bcl-2-associated agonist of cell death (Bad). This causes cytochrome c (Cyt C) to be released from the mitochondria and activates caspases, culminating in apoptosis. They also contribute to the activation of activator protein-1 (AP-1), a transcription factor located in the nucleus that controls the expression of pro-inflammatory cytokines. Other abbreviations: CAT, catalase; COX, cyclooxygenase; GPx, glutathione peroxidase; IL, interleukin; MS, methionine synthase; NOX, nicotinamide adenine dinucleotide phosphate oxidase; SOD, superoxide dismutase; TNF, tumor necrosis factor.

The occurrence of CVD in China is notably elevated, with a significant proportion of cases being linked to hyperhomocysteinemia (HHcy) [73]. Hcy can promote oxidative stress by facilitating the production of ROS, thereby initiating vascular inflammation (Figure 4). Oxidative stress and inflammation can therefore induce endothelial dysfunction and vascular stiffness, ultimately leading to the development of various CVD risk factors such as hypertension, kidney failure, and hyperlipidemia [74,75].

The most common cause of HHcy stems from an unbalanced dietary pattern. Folate, both natural and synthetic, has been reported to have beneficial effects on cardiovascular protection (Table 1). It is reported that administration of folate in a 2 mg/kg diet for 4 weeks significantly decreased the plasma Hcy levels, thereby achieving a 15 mm Hg reduction in the systolic blood pressure (BP) in spontaneously hypertensive rats (SHRs) [76]. In another study, the SHRs with HHcy receiving folate at a dose of 0.4 mg/kg/day for 8 weeks showed a reduction in oxidative stress, as evidenced by decreased malondialdehyde (MDA) levels and increased superoxide dismutase (SOD) levels [77]. In addition, the mRNA expression of IL-6 and NF-κB p65/Rela was decreased, demonstrating the anti-inflammatory properties of folate in SHRs. Similarly, Gao, et al. [78] reported a drop in serum MDA level and an increase in SOD level, and the expression level of NADPH oxidase 2 (NOX2) and NOX4 were downregulated in SHRs at the same level of administration. NOXs promote the production of ROS, uncoupling endothelial nitric oxide synthase (eNOS), which is responsible for producing NO, an important molecule to support cardiovascular homeostasis. However, data from a prospective cohort study launched in Wuhan, China, showed a positive correlation between the administration of a high-dose FA supplement (≥800 μg DFE/day) from pre-pregnancy to mid-pregnancy and an increased risk of developing gestational hypertension [79]. This dosage exceeds maternal RDA by only 200 μg, so pregnant women should exercise caution when consuming folate, both dietary folates and supplements.

Apart from spontaneous hypertension, folate can also treat hypertension caused by external hormones, such as angiotensin II. It is a peptide hormone that causes vasoconstriction and is commonly used for the treatment of hypotension, but it can also lead to hypertension and other side effects when its level remains high for a long period of time. Pushpakumar, et al. [80] revealed that supplementation of FA through drinking water effectively decreased the plasma Hcy level in angiotensin II-infused mice. This increased NO production, eNOS protein expression, renal blood flow, and vascular density, reduced ROS production, and ultimately resulted in reduced systolic BP and repaired renal injury. More recently, it was found that oral administration of FA (0.006% *wt*/*wt*) in a methionine diet for 3 weeks to mice prior to angiotensin II infusion could reverse cardiac hypertrophy by attenuating the expression of calcineurin and nuclear factor of activated T cells (NFAT) [81]. In addition, FA has shown protective effects on cardiac inflammation and fibrosis by inhibiting NF-κB and TGF-β signaling pathways.

Recent studies have revealed the significance of FA in mitigating hyperlipidemia, inflammation, and oxidative stress, which are major risk factors leading to the development of atherosclerosis, through various mechanisms (Table 1). Administering 75 mg/kg/day of FA to high-fat-fed LDL receptor-deficient (LDLR−/−) mice for 16 weeks resulted in a significant inhibition in atherosclerosis plaque progression and vascular smooth muscle cell (VSMC) dedifferentiation by modulating the mTOR/p70S6K signaling pathway, which involved the reduction in lipid levels and the suppression of oxidative stress and inflammatory response [82]. Similar results were also found in a recent study conducted by Zhong, et al. [83], who used the same apoE- murine model and found that the combination of FA supplementation (0.006%) and aerobic exercise decreased the aortic root plaque area and plaque burden, as well as the plasma monocyte chemoattractant protein-1 (MCP-1) level, a pro-inflammatory cytokine that increases the progression of atherosclerosis. Additionally, FA supplementation at doses of 5 mg/kg and 10 mg/kg has been shown to be successful in treating hyperlipidemia caused by liver cholestasis in rats with bile duct ligation (BDL) by elevating the glutathione level and lowering the Hcy, triglyceride, total cholesterol, high-density lipoprotein (HDL), and LDL levels [84].

Currently, there is a growing trend where FA is combined with fatty acids, vitamins, and pharmaceuticals to achieve cardioprotective effects. It was found that a combined intake of FA, vitamin B12, and n-3 fatty acids (DHA + EPA) lowered the Hcy, MDA, and placental tumor necrosis factor a (TNF-α) levels, leading to a decrease in oxidative stress and systolic BP in rats with pregnancy-induced hypertension [85]. However, individual supplementation of FA did not show positive effects in this study. Zhu, et al. [86] reported that the combination of losartan and FA could smooth the arterial endothelial cells and improve the integrity of the cellular membrane of SHRs.

### 5.2. Hematopoiesis Protection

Folate also has significant protective activity for hematopoiesis, which is reflected in its participation in DNA synthesis [87], assistance in hemoglobin production [88], and maintenance of the normal metabolism of hematopoietic cells [89]. Folate plays a role in various metabolic reactions such as amino acid metabolism and DNA methylation, which is conducive to the proliferation, differentiation, and functional maintenance of hematopoietic cells [90]. However, some research studies have also shown that both folate deficiency and excessive dietary folate levels compromise hematopoiesis, resulting in defective cell cycle progression, persistent DNA damage, and impaired production of lymphocytes [91]. If there is a deficiency of folate, it will lead to the obstruction of DNA synthesis, and increase the volume of cells but prevent them from maturing, thereby causing megaloblastic anemia. The participation of folate can ensure the normal synthesis of hemoglobin, thereby enhance the oxygen-carrying capacity of red blood cells and prevent the aggravation of anemia symptoms due to an insufficient hemoglobin content.

Folate interacts with other vitamins and minerals in the process of protecting hematopoiesis. For instance, vitamin B12 and folate jointly participate in DNA synthesis and cellular metabolism in the body [92]. The combination of folate and vitamin B12 is significantly better than taking either folate or vitamin B12 alone [93]. Folate can promote the absorption and utilization of iron, and at the same time, iron can also provide the necessary raw materials for the synthesis of hemoglobin in which folate participates [92]. Vitamin C can reduce folate to tetrahydrofolate, promoting its absorption and utilization. The antioxidant effect of vitamin C can protect folate from oxidative damage, enhancing its stability and effectiveness [93]. Zinc is involved in the regulation of enzyme activity in folate metabolism and indirectly affects hematopoietic function [94].

### 5.3. Gut Homeostasis Maintenance

While there have been several studies demonstrating the efficacy of folate in managing various chronic conditions such as cancer, neurodegenerative diseases, and type II diabetes mellitus, the exploration of the impact of folate on gut homeostasis remains relatively limited. Gut homeostasis is maintained through the interaction of various elements and mechanisms that encompass the gut microbiome, gut barrier, and gut immune system [95]. The gut microbiome comprises a diverse community of microorganisms, including bacteria, fungi, and viruses, which play a crucial role in maintaining host health, producing essential vitamins, and defending against pathogens [96]. Although a certain amount of folate can be synthesized in vivo by the gut microbiome, a folate-deficient diet can cause significant shifts in the diversity of the gut microbiome. This, in particular, leads to a decrease in the abundance of *Bifidobacterium* and *Lactobacillus*, two pivotal bacteria that depend on folate for metabolism and produce folate, ultimately affecting the efficacy of folate biosynthesis [97,98]. Wang, Zou, Li, Yang and Yin [12] examined the effect of FA on the composition of the gut microbiome and the content of short-chain fatty acids (SCFAs) in weaned piglets. The results showed that there was an increase in the abundance of *Lactobacillus* in the caecum of piglets, and the increase was found to be positively correlated with the content of acetic acid; meanwhile, a higher production of SCFAs, including isobutyric acid, butyric acid, and isovaleric acid, was observed in the colon than in the caecum. Liu, et al. [99] demonstrated that broiler chicken fed with 13 mg/kg FA for 4 weeks showed reduced fat deposition by downregulating genes associated with adipocyte proliferation and differentiation, facilitated by alterations in the composition of gut bacteria and the generation of SCFAs. In particular, the levels of acetic acid, propionic acid, and isobutyric acid were noticeably elevated. SCFAs are primary metabolites that are synthesized by the gut microbiome through the fermentation of dietary fiber or complex carbohydrates [100]. These SCFAs are capable of eradicating certain pathogens belonging to the Enterobacteriaceae family, including Escherichia coli and Salmonella, by lowering the pH in the intestinal lumen [101]. Nevertheless, different forms of FA can have distinct effects on community richness, gut microbiome composition, and SCFA profiles. In a human fecal slurry culture model, it was found that the Ace index in the FA group was higher than that of the 5-MTHF group [102]. Furthermore, although most changes in gut microbiota abundance were identical in both groups (e.g., *Aetinobacteriota* and *Bacteroidota*), there was a decrease in the abundance of the Firmicutes phylum in the FA group and an increase in the 5-MTHF group, which may differentially affect body weight and inflammatory responses. In contrast, Mir, et al. [103] observed significant alterations in the colonic mucosal bacteria composition, possibly predisposing the microbiome to colitis, across multiple taxonomic levels, resulting from the administration of methyl donor micronutrients during pregnancy in a murine model, and these changes were independent of any postnatal maternal exposures. This implies that extended periods of folate supplementation potentially have negative effects on the gut microbiome composition.

It has been found that increased abundance of *Enterococcus*, *Lactobacillus*, and *Bifidobacterium* species in the gut can promote the production of neurotransmitters such as γ-aminobutyric acid (GABA), serotonin, dopamine, and acetylcholine, which are closely correlated with the gut microbiota–brain axis and help regulate anxiety and depression [104]. A study conducted by Zhou, et al. [105] found that FA (0.8 mg/kg for 6 weeks) was able to increase the levels of dopamine and norepinephrine in serum and brain tissue of rats. Nevertheless, the link between folate and the gut microbiota–brain axis warrants further investigation to fully understand the mechanisms by which folate influences neurotransmitter production and mental health.

In addition to gut microbiome composition, folate has been shown to maintain the integrity of the gut barrier. The intestinal barrier consists of several layers, with the outermost layer consisting of the mucus layer, commensal gut microbiome, and defense proteins, followed by intestinal epithelial cells (IECs) in the middle layer, and the innermost layer comprising immune cells [106]. Among these constituents, IECs are closely related to the gut microbiome, acting as a dynamic interface that bridges the host immune system and the microbial community, and the well-being of both IECs and the gut microbiome holds significant importance. Briefly, the IECs facilitate the establishment of a conducive environment for commensal microorganisms by secreting mucins, producing antimicrobial peptides, and absorbing microbial metabolites; in turn, the gut microbiome blocks the passage of detrimental substances such as pathogenic bacteria, toxins, and luminal antigens from the intestinal lumen into the bloodstream [107]. There are numerous factors affecting the gut barrier integrity, including infection, inflammation, a dysregulated immune response, a low-fiber dietary pattern, and stress. A compromised gut barrier has been linked to several chronic and systemic diseases, particularly inflammatory bowel disease (IBD), CVD, non-alcoholic fatty liver disease, and type I diabetes mellitus [108]. Zhu, et al. [109] administered FA at 0.071 mg/kg to rats with dextran sulfate sodium (DSS)-induced colitis for 7 days and found that the pro-inflammatory pathways were suppressed, indicating that FA can be a potential treatment option for IBD exacerbated by HHcy. A cross-sectional study also revealed a notable increase in the abundance of Akkermansia, a probiotic associated with the potential regulation of weight loss and inflammation, in a population with high dietary folate consumption (≥227 mg DFE/d) [110]. In addition, a 25-week intervention of FA in mice fed a high-fat diet at 5 mg/kg was effective in alleviating obesity by altering the composition of gut microbiota [111]. Specifically, the abundance of *Allobaculum* and *Rekenella*, which have health-promoting functions such as improving gut barrier integrity and regulating inflammation, was increased, while the abundance of *Oscillibacter* and *Bilophila*, which produce LPS and thereby influence gut permeability and adiposity, was decreased. A similar pattern of alteration in the gut microbiota composition was found by Sun, et al. [112], who demonstrated that FA was effective in mitigating hyperuricemia by reducing the firmicutes-to-bacteroidota ratio, a crucial marker for intestinal homeostasis.

### 5.4. Immune Response Enhancement

Although the gut barrier and the immune system are closely intertwined and contribute to gut homeostasis, they have their own distinct roles. The gut barrier serves as a first physical safeguard, while the gut immune system is responsible for immunological defense and the maintenance of an equilibrium between protection and tolerance towards probiotics and other substances [113]. Any malfunction in either component can result in a wide range of gastrointestinal diseases and immune system disorders. Akinyemi and Adewole [114] found that supplementing broiler chickens with increased levels of FA (2.2–15 ppm) may aid in mitigating immune damage induced by the inflammation arising from high-energy diets by increasing the bursa weight, a reliable immune system health indicator. A study conducted by Gouda, et al. [115] revealed that after the administration of FA in 1.5 mg/kg in the diet for 5 weeks, there was a substantial enhancement in the immune response towards Newcastle disease virus in boiler chickens, a highly transmissible and possibly fatal virus that mostly affects birds. Similar results were reported by Lin, et al. [116], who demonstrated that the non-specific immune responses responded positively to dietary FA supplementation after an 8-week growth trial in fish. In another study, broilers were injected with different doses of FA (0–150 mg) at embryonic age 11 d, and the results showed that the plasma IgG, IgM, and lysozyme activity was increased, and the splenic expression levels of IL-2 and IL-4 were up-regulated [117]. In addition, it was found that administering FA (0.3–15 mg/kg) to weanling piglets enhanced the serum IFN-g level and lowered the CD3^+^ CD4^+^/CD3^+^ CD8^+^ ratio from 1.57 to 1.07, which facilitated effective activation of immune cells [13]. Previous studies also suggested that FA is essential for maintaining the level of Foxp3^+^ regulatory T cells in the intestine of mice, which are a subset of CD4^+^ T cells that are responsible for orchestrating immune responses by activating and modulating other immune cells [118,119]. Nevertheless, compared to the intestine, the effect observed in the spleen and the mesenteric lymph node was less pronounced. This could be attributed to the unique gut microenvironment, which is consistently exposed to foreign substances originating from both dietary sources and commensal microbes, allowing regulatory T cells to remain in a highly activated state. Nevertheless, the study conducted by Meadows, et al. [120] demonstrated that a diet rich in FA (10 × recommended level) could enhance parasite multiplication, impair immune responses, and diminish resistance to malaria in a murine model. These results emphasized the importance of recognizing the potential downsides linked to the elevated dietary folate consumption on the immune system.

### 5.5. Neuroprotection

Neurodegenerative disorders encompass a collection of chronic progressive conditions that predominantly impact the structure and function of the central nervous system, with prevalent examples being Alzheimer’s disease, Parkinson’s disease, Huntington’s disease, and amyotrophic lateral sclerosis [121]. Folate may enhance cognitive function by modulating the expression of biomarkers associated with neurodegenerative processes. Amyloid-b (Ab) is a peptide that aggregates and forms plaques in the brains of individuals with Alzheimer’ disease; hence, a range of Ab-related biomarkers can aid in the early detection and tracking of the progression of Alzheimer’s disease [122]. A randomized controlled trial showed that individuals with mild cognitive impairment who received 400 mg/day of FA for 24 months had enhanced cognitive function, as evidenced by improved scores on the full-scale IQ, verbal IQ, information, and digit span tests; in addition, blood levels of Ab-related biomarkers, Ab-42 and APP-mRNA, were reduced [8]. Similarly, Zhao, et al. [123] reported that mice fed with FA at 2 mg/kg for 25 weeks showed an improved performance in the open field test, elevated plus maze, and Morris water maze, suggesting the efficacy of FA in reducing anxiety-related activity and enhancing learning and memory capacity. Zhu, et al. [124] investigated the protective effect of FA related to miR-19 against aluminum-induced neural cell apoptosis and suggested that FA could down-regulate miR-19 by modulating the expression of a range of apoptotic proteins, including PTEN, p-AKT, p53, Bax, Bcl-2, caspase-9, and caspase-3. Another study reported by Li, et al. [125] found that FA inhibited apoptosis in astrocytes by promoting cell proliferation, decreasing Hcy and ROS levels, and suppressing telomere shortening and telomeric DNA oxidative damage. Moreover, it was found that the combination of FA (40 mg/mL) and genistein (27 mg/mL) in the treatment of Ab31-35-exposed rat cortical neuron cultures showed an enhanced neuroprotective effect by down-regulating the apoptotic-related genes Bax, p53, and caspase3 and up-regulating Bcl-2 [126].

A number of studies have provided evidence indicating that the folate status may have an impact on the onset or alleviation of neurodegenerative disorders through several other mechanisms, including Hcy levels, methylation processes, inflammation, and oxidative stress (Table 1). Li, et al. [127] demonstrated that FA could increase DNA methylation in N2a-APP cells and PS1-APP mice by down-regulating the mRNA expression of genes in the JAK-STAT and LTD pathways. Another study investigated the potential effect of FA on cerebellar damage induced by Hcy in Wistar rats and found that FA effectively reduced this neurotoxicity by decreasing the Hcy level and lipid peroxidation and increasing motor coordination and glutathione peroxidase (GPx) activity [128]. According to Zhao, et al. [129], the combined supplementation of FA and soy isoflavone has been shown to improve neuroprotection, as demonstrated by increasing antioxidant activity as well as reducing neuron apoptosis and DNA damage. In addition, a randomized controlled trial recruiting participants with newly diagnosed Alzheimer’s disease who consumed 1.25 mg/day of FA for 6 months showed that the intervention group had significantly higher scores on the mini-mental state examination, which may be associated with reduced levels of inflammation biomarkers TNF-α and TNF-α-mRNA as well as Ab-related biomarkers Ab-40 and PS1-mRNA [130].

Although elevated folate levels have shown their potential in mitigating neurodegenerative disorders, it is crucial to acknowledge that excessively high folate levels may induce various adverse effects in individuals. A clinical trial examined the effectiveness and safety of administering a high dosage of folate (5000 mg/d) in combination with vitamins B6 and B12 in the treatment of mild to moderate Alzheimer’s disease, and their findings indicated that the treatment did not have a positive effect in slowing cognitive decline in the affected individuals and revealed an increased incidence of adverse events, including depression, in the participants [131]. In another study conducted by Bailey, et al. [132], individuals with normal vitamin B12 levels were found to have a reduced risk of experiencing cognitive impairment, whereas higher levels of folate seemed to have a protective effect; conversely, in cases where the level of vitamin B12 was deficient, higher levels of folate were found to be substantially correlated with a worse cognitive performance across many assessments. Additional research is needed to elucidate the underlying mechanisms by which folate interventions work in combination with other B vitamins in order to ensure their safety and determine the optimal dosage.

### 5.6. Anti-Cancer Activity

Cancer is a major global public health issue, characterized by the unregulated proliferation and dissemination of abnormal cells in the body, with prevalent forms including breast, lung, colorectal, and prostate cancers. Currently, surgery, radiation therapy, and chemotherapy have traditionally served as the fundamental pillars of cancer treatment. However, ongoing efforts persistently investigate novel alternative approaches considering the recently identified limitations associated with the aforementioned treatment methods. For instance, chemotherapy is non-specific, which means it can affect both cancerous and healthy dividing cells, leading to serious side effects such as nausea and fatigue [133]. Folate, a vitamin involved in DNA methylation and a variety of cellular processes, has garnered much attention in contemporary research. Maintaining genomic integrity through appropriate DNA methylation patterns is crucial for hindering the growth of cancer cells, as aberrant DNA methylation can activate oncogenes and silence tumor suppressor genes [134].

Folate has the capacity to impede the development of cancer cells by virtue of its involvement in one-carbon metabolism, which encompasses the regeneration of methionine from Hcy, the synthesis of S-adenosylmethionine (SAM), the regulation of DNA methyltransferase, and the facilitation of thymidine synthesis [135]. In an in vitro study using MCF-7 cells, it was found that when exposed to FA at a concentration of 4 mg/L, the expression of three tumor suppressor genes, PTEN, APC, and RARb2, was upregulated by causing hypermethylation in their promoters [11]. Additionally, when the concentration of FA was doubled to 8 mg/L, there was an observed increase in the expression of DNA methyltransferase 1 (DNMT1). A cross-sectional study conducted among elderly Chileans also demonstrated that having a high physiologic folate level (≥45.3 nmol/L) increased the methylation at the third tertile of specific CpG sites in tumor suppressor gene p16, as well as in the DNA repair genes MLH1 and MGMT [136]. Wang, Pan, Su, Huang, and Sun [10] examined the relationship between folate and DNA methylation of p16 and p53 in individuals with esophageal precancerous lesions and esophageal squamous cell carcinoma. The results showed that a high serum folate level, in conjunction with unmethylated gene promoter regions, was associated with a reduced risk of esophageal cancer, and the high serum folate level could counteract the tumor-promoting effects resulting from aberrant DNA methylation.

Apart from its role in DNA methylation, folate exhibits antioxidant properties that have been found to regulate the proliferation of cancer cells through three main mechanisms: (i) enhances the coupling of eNOS to cofactor tetrahydrobiopterin (BH4), hence inhibiting the formation of superoxide anion and the resulting peroxynitrite, a potent oxidant involved in various pathological conditions; (ii) facilitates the generation of reduced glutathione, an amino acid functioning as an endogenous antioxidant, and is crucial in neutralizing ROS and safeguarding cellular integrity; (iii) re-methylates Hcy, a risk factor associated with increased vulnerability to oxidative stress, to methionine [137,138]. FA was also shown to possess anti-genotoxic properties in HT-29 cells, as demonstrated by a reduction in micronucleus and comet tail DNA [139]. However, according to a Prostate, Lung, Colorectal, and Ovarian Cancer Screening Trial cohort study conducted by Stolzenberg-Solomon, et al. [140], women who consumed ≥400 mg of supplemental FA per day had a 19% higher risk of developing postmenopausal breast cancer than those who did not consume supplemental FA. Furthermore, it has been observed that delivering a high-dose FA supplement to mice with early colonic lesions could facilitate the formation of cancer, suggesting that the FA supplement could promote CRC in individuals with IBD if administrated at high doses [141]. Therefore, it is important to consider the potential risk factors of FA supplementation, especially in specific populations such as pregnant women.

### 5.7. Neural Tube Protection

The neural tube is an anatomical structure that arises during the early stages of embryogenesis in humans, ultimately giving rise to the central nervous system, including the brain and spinal cord. When the neural tube fails to close completely during that stage, it results in a group of congenital disorders in the embryo referred to as NTDs, with spina bifida and anencephaly being the two most common examples [142]. The causes of NTDs include intricate interplays between genetic predisposition and environmental and maternal factors, and a common factor contributing to the elevated risk of NTDs is maternal folate deficiency [143].

Although the role of folate in NTDs is well known, recent studies have further revealed its mechanism of action. According to a systematic study, the prevalence of NTDs varies between 0.69 and 2.19 per 1000 live births across different regions [144]. Adequate amounts of folate are necessary to support nucleotide synthesis and facilitate rapid cell division and differentiation, processes that are crucial for the closure of the neural tube. Cawley, et al. [145] revealed that a daily maternal intake of 400 μg of FA, especially when started before conception and continued through the early stages of pregnancy during neural tube closure, resulted in optimal RBC folate levels (906 nmol/L), a reliable indicator of long-term folate status. FA supplementation was found to reduce methotrexate-induced histone H2A monoubiquitylation and upregulate the expression of neural tube closure-related genes, including Cdx2, Nestin, Pax6, and Gata4, subsequently reestablishing the binding of H2A monoubiquitylation with these genes [146]. According to Zhao, et al. [147], FA supplementation at 3 mg/kg/day significantly alleviated LPS-induced NTDs in mice due to its ability in mitigating GSH depletion, JNK phosphorylation, and NF-κB activation. Similar results were reported by Gao, et al. [148], who found that FA attenuated NTDs induced by hyperglycemia in pregnant mice, which might be related to the reduction in oxidative stress as well as the restoration of Pax3 expression. In addition, the down-regulation of p53, NF-κB, Pim-1, and c-Myb expression, along with the Bax/Bcl2 ratio in mice, suggested that the preventive effect of FA on NTDs was also linked to its anti-apoptotic effect [149]. However, the administration of a high dosage of FA supplement (>1000 mg) did not yield a higher absorption rate or provide protection against recurrent NTDs, so it is wrong to assume that more is always better when it comes to FA supplementation [150]. The proper course of action is to follow the recommended dosage and consult with a healthcare provider before increasing the intake beyond what is advised.

The synergistic effects between folate and other nutrients, including n-3 fatty acids, flavones, and multivitamins, were extensively investigated in the prevention of NTDs in a wide range of animal studies and human interventions (Table 1). In a murine model with diabetes-induced NTDs, the combined intake of FA and n-3 PUFA significantly lowered the Hcy level and NTD incidence by attenuating Pax3 hypermethylation and modulating the expression levels of genes involved in one-carbon metabolism [151]. In another study, FA was supplemented with soybean isoflavone to pregnant rats, and induced by cyclophosphamide, and the results showed that the occurrence of NTD was significantly decreased [129]. Interestingly, an evidence-based model established by Kancherla, et al. [152] suggested that the introduction of mandatory FA fortification of salt at 20 ppm was able to prevent about 65% of annual cases of FA-preventable spina bifida and anencephaly worldwide. More recently, there has been a belief that excessive FA supplementation can mask vitamin B12 deficiency; nevertheless, the consumption of folate from natural and fortified foods often falls short of recommended levels, especially for individuals following a low-carbohydrate diet [16]. Hence, pregnant women are suggested to incorporate micronutrient supplements into their dietary regimen, as this practice not only reduces birth defects but also provides adequate nutrients to maintain maternal health.

### 5.8. Danger of Excess Folate and Recommendations for FA Intake

Although adequate FA supplementation provides multiple health benefits in the fields of nutrition and medicine, it is important to acknowledge that excessive intake poses significant hidden dangers. Excessive intake of FA may have multiple negative impacts on health. During pregnancy, although moderate FA supplementation is crucial for preventing neural tube defects, excessive intake of FA may lead to adverse pregnancy outcomes, such as a low birth weight, shortened birth length, increased risk of migraines, and elevated risk of gestational diabetes, especially when there is an imbalance between high FA and vitamin B12 levels (Fardous, A.M. et al., 2023) [153]. In addition, excessive FA may also affect the long-term health of offspring through epigenetic changes, increasing the risk of insulin resistance, obesity, and autism spectrum disorder in the next generation [153]. In terms of neurodevelopment, excessive FA supplementation is associated with impaired neurocognitive development in children, which may lead to poor psychomotor development and an increased risk of autism [153]. In terms of immune function, excessive FA may reduce the cytotoxicity of natural killer cells and increase the risk of asthma and other allergic diseases in children. In terms of carcinogenic effects, excessive FA may increase the risk of cancer in the presence of precancerous lesions, especially in rapidly proliferating precancerous or transformed cells [153]. In addition, high FA intake may also be associated with a slight increase in all-cause mortality, although this conclusion still requires further research for verification. Therefore, although FA has many benefits for health, excessive intake may bring a series of potential risks. It is recommended to follow the recommended intake to avoid adverse health effects [154]. In addition, high-concentration FA intake has been found to be associated with interactions with epilepsy medications, masking vitamin B12 deficiency and hepatotoxicity [155]. Therefore, maintaining a healthy level of FA intake is crucial.

The Recommended Dietary Allowance (RDA) for folate varies across populations (Table 2): Adults require 400 µg/day of dietary folate equivalents (DFEs), while children have age-specific requirements (150 µg DFE for 1–3 years; 200 µg DFE for 4–8 years; 300 µg DFE for 9–13 years; 400 µg DFE for 14–18 years) [156]. Pregnant and lactating women require higher intakes of 600 µg DFE and 500 µg DFE, respectively. For infants aged 0–6 months and 7–12 months, the adequate intake levels are 65 µg DFE and 80 µg DFE, respectively [156].

The Tolerable Upper Intake Level (UL) for folate is established solely for synthetic FA from supplements due to its interaction with vitamin B12. Daily supplementation of FA ≥5000 µg may correct the megaloblastic anemia caused by vitamin B12 deficiency, potentially masking and delaying its diagnosis. Consequently, high FA intake can allow the progression of irreversible neurological complications associated with untreated B12 deficiency. For this reason, the Institute of Medicine (IOM) has set a UL of 1000 µg/day for FA.

**Table 1:** Health benefits of folate reported from in vivo and in vitro studies and human interventions.

Model/object	Dosage and Duration	Outcomes	Reference
Cardiovascular protection			
SHRs	2 mg/kg diet for 4 weeks	Systolic BP ↓; plasma Hcy ↓; liver TG ↓; glucose tolerance ↑; insulin resistant ↑; oxidative stress (SOD ↑; GPx ↑; GSH ↑; GSSG ↑; TBARS ↓)	[76]
Ang II-infused mice	0.015 g/L in drinking water	Plasma Hcy ↓; systolic BP ↓; oxidative stress (ROS production ↓); renal function (renal cortical blood flow ↑; vascular density ↑; Nox2/Nox4 ↓; eNOS ↑; ADMA ↑; VEGF ↓; Col IV ↓)	[80]
SHRs	0.4 mg/kg/d for 8 weeks	Hcy ↓; oxidative stress (SOD ↑; MDA ↓); inflammation (IL-6 ↓; NF-κB p65/Rela ↓)	[77]
SHRs	0.4 mg/kg/d for 8 weeks	Plasma Hcy ↓; oxidative stress (serum SOD ↑; serum MDA ↓); renal function (NOX2/NOX4 ↓; UACR ↓; GFR ↑; glomerular sclerosis index ↓)	[78]
Ang II-infused mice	0.006% wt/wt for 3 weeks	Systolic BP ↓; cardiac hypertrophy (calcineurin ↓; NFAT ↓; heart wt/body wt ↓; contractility ↓; myocyte area ↓; ANF ↓; BNP ↓); inflammation (CD68^+^ area ↓; IL-6 ↓; IL-1b ↓; TNF-α ↓, NF-κB ↓; TGF-β ↓); fibrosis (a-SMA ↓; Col I ↓; Col III ↓)	[81]
LDLR^−/−^ mice with high-fat diet	75 μg/kg/d for 16 weeks	TG ↓; TC ↓; LDL ↓; VLDL ↓; HDL ↑; oxidative stress (serum SOD ↑; serum MDA ↓; serum GPx ↑); inflammation (IL-6 ↓; IL-1β ↓; TNF-a ↓); atherosclerotic lesion area ↓; VSMC dedifferentiation (a-SMA ↑; OPN ↓)	[82]
ApoE^−/−^ mice	0.006% wt/wt (combined with aerobic exercise) for 8 weeks	Plasma Hcy ↓; atherosclerosis (aortic root plaque area/burden ↓; plasma MCP-1 ↓)	[83]
BDL rats	5 and 10 mg/kg for 4 weeks	Serum Hcy ↓; oxidative stress (GSH: GSSG ↑); TG ↓; TC ↓; HDL ↑; LDL ↓	[84]
Pregnancy-induced hypertension in rats	8 mg/kg diet (combined with 120 mg of DHA, 180 mg of EPA, and 50 mg of VB_12_) for 20 d	Dam: Systolic BP ↓; plasma Hcy ↓; oxidative stress (plasma MDA ↓); inflammation (placental TNF-α↓); placental AA ↓ Offspring: oxidative stress (liver MDA ↓; liver protein carbonyl ↓); liver DHA ↑	[85]
SHRs	0.4 mg/kg/d (combined with losartan at 25 mg/kg/d) for 12 weeks	More smooth and intact cellular membrane of endothelial cells	[86]
Mice with triple-transgenic late-stage Alzheimer’s disease	12 mg/kg/d for 3 months	Cardiac apoptosis (apoptotic cells ↓; fas-ligand ↓; FADD ↓; BAK ↓; cytochrome-c ↓; cleaved-caspase-3 ↓); p-IGF1-receptor ↑; p-PI3K ↑; p-AMPKα ↑; sirtuin 1 ↑	[9]
Hematopoiesis protection			
Mice	2 mg/kg for 6 months	Higher hematopoietic reconstitution potential and numbers of circulating lymphocytes	[91]
Zebrafish model of congenital sideroblastic anemia	100 mM glycine with 1 mM sodium for 44 h	Higher hemoglobin levels	[88]
Human colon epithelial cells	Presence or absence of FA (4 mg/L) for up to 14 d	Folate deficiency increases uracil misincorporation two- to threefold in these cells Folate-deficient human colonocytes exposed to hydrogen peroxide or methyl methane sulfonate, an alkylating agent, are unable to repair DNA strand breakage as efficiently as folate-sufficient cells	[87]
240 participants with MCI	FA alone, vitamin B12 alone, FA plus vitamin B12, or control without treatment daily for 6 months	Post hoc Turkey tests found that FA and vitamin B12 supplementation was significantly more effective than FA alone for all endpoints	[8]
C2C12 myoblast cells	Vitamin C at 100 µM	Compared to untreated cells, treatment of C2C12 cells with AA at 100 µM resulted in enhanced concentrations of FA (2.5-fold) and 5-MTHF (10-fold increase)	[93]
Patients undergoing hemodialysis	ESA consisted of darbepoetin alfa (injection at doses of 10, 20, 30, and 40 μg) HIF-PHI included roxadustat (tablets at doses of 20, 50, and 100 mg)	Zinc supplementation improves ERI during darbepoetin alfa administration. The use of roxadustat stabilizes HIF-1α, HIF-2α, and HIF-3α	[94]
Gut homeostasis maintenance			
Piglets	3–18 mg/kg for 14 d	Caecum (pH ↓; acetic acid ↑; *Firmicutes* ↑; *Lactobacillus reuteri* ↑; *Lactobacillus salivarius* ↑; *Lactobacillus mucosae* ↑); colon (IBA ↑; BA ↑; IVA ↑; acetic acid/propionic acid ↑; total SCFAs ↑)	[12]
Mice with DSS-induced colitis	0.071 mg/kg for 7 d	Plasma Hcy ↓; inflammation (p-p38 ↓; p-cPLA-2 ↓; COX-2 ↓; PGE2 ↓; IL-17 ↓; RORgt ↓)	[109]
Male veterans for colonoscopy procedures	≥227 μg vs. <227 μg DFE/d	*Firmicutes* (*Dialister* ↑; *Roseburia* ↑; *Faecalibacterium* ↑); *Verrucomicrobia* (*Akkermansia* ↑); *Bacteroidota* (*Bacteroides* ↓; *Alistipes* ↑; *Odoribacter* ↑; *Parabacteroides* ↑)	[110]
Mice fed with high-fat diet	5 mg/kg for 25 weeks	Body weight ↓; Ace index ↑; *Firmicutes* ↓ (*Streptococcus* ↑; *Colidextribacter* ↓; *Allobaculum* ↑; *Lactococcus* ↑; *Oscillibacter* ↓; *Roseburia* ↓; *Tuzzerella* ↓; *Ileibacterium* ↑); *Bacteroidota* ↑ (*Rikenella* ↑); *Desulfobacterota* (*Desulfovibrio* ↓; *Bilophila* ↓)	[111]
Rats fed with high-purine diet	4 mg/kg for 8 weeks	Uric acid ↓; *Firmicutes* ↓ (*Lactobacillus* ↑; *Clostridium* ↓; *Romboutsia* ↓; *Blautia* ↑; *Ruminococcus* ↓); *Actinobacteria* ↑ (*Collinsella* ↑); *Desulfobacterota* ↓; *Bacteroidota* ↑ (*Bacteroides* ↑)	[112]
Broiler chicken	13 mg/kg for 4 weeks	Abdominal fat percentage ↓; *Firmicutes* ↓ (*Clostridium* ↑; *Oscillospira* ↑; *Ruminococcus* ↑; *Dehalobacterium* ↑); *Bacteroidota* ↑ (*Alistipes* ↑; *Parabacteroides* ↑); cecal (acetic acid ↑; propionic acid ↑; IBA ↑); adipocyte proliferation and differentiation genes (IGF1 ↓; EGF ↓; TGF-β ↓; C/EBPα ↓; FABP-4 ↓; PCNA ↓)	[99]
Human fecal slurry culture	0.5 mL of fecal suspension into 100 μL of 1 mg/mL FA or 5-MTHF for 24 h fermentation	FA: Ace index ↑; Shannon index ↓; acetic acid ↓; *Firmicutes* ↓ (*Lactobacillus* ↑; *Pediococcus* ↑); *Proteobacteria* ↑; *Actinobacteriota* ↑ (*Bifidobacterium* ↑); *Bacteroidota* ↓ (*Bacteroides* ↓) 5-MTHF: acetic acid ↓; Shannon index ↓; *Firmicutes* ↑ (*Lactobacillus* ↑; *Pediococcus* ↑); *Actinobacteriota* ↑ (*Bifidobacterium* ↑); *Bacteroidota* ↓ (*Bacteroides* ↓)	[102]
Immune response enhancement			
Broiler chicken	1.5 mg/kg diet for 35 d	Thyroid hormones (IGF-1 ↑; triiodothyronine ↑; thyroxin ↑); oxidative stress (HSP70 ↑; TAC ↑; catalase ↑; SOD ↑); immune response (H/L ↓; antibody titration against NDV ↑)	[115]
Fish (*Epinephelus malabaricus*)	0–10 mg/kg diet for 8 weeks	Oxidative stress (TBARS ↓; SOD ↑; superoxide anion production ↑); immune response (lysozyme ↑)	[116]
BALB/c mice	4 μg/mL in drinking water	Colon immune response (Foxp3^+^ CD4^+^ ↑; IFN-g^+^ CD4^+^ ↑; IL-10 ↑; FR4^+^ CD4^+^ ↑; Bcl-2/GAPDH ↑; Bcl-xL/GAPDH ↑)	[118]
Female BALB/c mice	Control diet for 8 weeks	Immune response (small intestinal Foxp3^+^ CD4^+^ ↑)	[119]
Broiler chickens with high-energy diet	2.2–15 ppm for 6 weeks	Ceca weight ↓; bursa weight ↓	[114]
Castrated weanling piglets	0–15 mg/kg diet for 24 d	Immune response (serum IFN-g ↑; CD3^+^ CD4^+^/CD3^+^ CD8^+^ ↓)	[13]
Fertilized broilers eggs	0–150 μg at embryonic age 11 d	Broiler chicks: one-carbon metabolism (MTHFR ↑; MTRR ↑); immune response (plasma lysozyme activity ↑; plasma IgG ↑; plasma IgM ↑; splenic IL-2 ↑; splenic IL-4 ↑; splenic IL-6 ↓)	[117]
Neuroprotection			
Patients with mild cognitive impairment	400 μg/d for 24 months	Hcy ↓; neurological test scores (full scale IQ ↑; verbal IQ ↑; information ↑; digit span ↑); Ab-related biomarkers (Ab-42 ↓; APP-mRNA ↓)	[8]
Rat cortical neuron cultures exposed to Ab31-35	40 mg/mL (combined with 27 mg/mL of genistein)	Neuron viability ↑; MMP ↑; DNA damage (comet cells ↓; DNA migration length ↓); apoptosis (*Bax* ↓; *Bcl-2* ↑; caspase-3 ↓; tumor p53 ↓)	[126]
Wistar rats with Hcy-induced cerebellar damage	0.011 mmol/g for 3 weeks	Plasma Hcy ↓; cortical Hcy ↓; motor coordination impairment ↓; oxidative stress (cerebellar MDA ↓; cerebellar GPx ↑)	[128]
ICR mice	2 mg/kg for 25 weeks	Hcy ↓; blood glucose ↓; serum insulin ↑; serum TG ↓; serum VLDL-cholesterol ↓; cognitive behaviors (open field test (peripheral time ↓; grooming ↓); elevated plus maze (open arm distance ↑; open/total arm entries ↑; open/total arm distance ↑); Morris water maze (latency ↓))	[123]
Patients with newly diagnosed Alzheimer’s disease	1.25 mg/d for 6 months	MMSE ↑; inflammation (TNF-α ↓; TNF-α-mRNA ↓; SAM ↑); Ab-related biomarkers (Ab-40 ↓; PS1-mRNA ↓;)	[130]
Mouse neuroblastoma N2a cells expressing human APP695	2.8–20 mmol/L	Increasing DNA methylation by down-regulating the mRNA expression of genes in JAK-STAT and LTD pathways	[127]
APP/PS1 mice	2.1 mg/kg diet + 600 mg/kg for 60 d	Increasing DNA methylation by down-regulating the mRNA expression of genes in JAK-STAT and LTD pathways	[127]
Human SH-SY5Y cells with Al-maltolate	10 mmol/L for 3 d	Cell viability ↑; miRNA-19 ↑; apoptosis (PTEN ↓; p-AKT ↑; p53 ↓; Bax ↓; Bcl-2 ↑; cleaved-caspase 9 ↓; cleaved-caspase 3 ↓)	[124]
Primary rat astrocyte culture	0–40 mmol/L for 12 d	Cell proliferation ↑; apoptosis ↓; Hcy ↓; ROS production ↓; telomeric DNA oxidative damage ↓; telomere length ↑	[125]
Wistar rats with cyclophosphamide-induced NTD	0.7 mg/kg (combined with 160 mg/kg of soy isoflavone) for 14/20 d	DNA damage (comet cells ↓; DNA migration length ↓); oxidative stress (SOD ↑; MDA ↓; NO ↓); apoptosis (p53 ↓; Bax ↓; Bcl-2 ↑)	[129]
Anti-Cancer activity			
200 OSCC cases, 200 OPL cases, and 200 control cases	<24.43, 24.43–29.14, 29.14–36.24, and >36.24 mg/L	OPL: risk ↓; p16 methylation ↓; p53 methylation ↓ OSCC: risk ↓; p16 methylation ↓; p53 methylation ↓	[10]
MCF-7 cells	4 or 8 mg/L	MCF-7 cell viability ↓; caspase-dependent apoptosis ↑; tumor suppressor genes (PTEN ↑; RARB2 ↑; APC ↑); DNMT1 ↑	[11]
HT-29 and SW480 cells	0, 100, 10,000 ng/mL	Micronucleus score ↓; comet tail DNA ↓	[139]
Elderly Chileans	<45.3 nmol/L and ≥45.3 nmol/L	Gene methylation (p16 ↑; MLH1 ↑; MGMT ↑)	[136]
Neural tube protection			
Mouse embryo stem cells	50 mg/L	Neural tube closure-related genes (Cdx2 ↑; Nestin ↑; Pax6 ↑; Gata4 ↑)	[146]
Women at their first antenatal visit	400 mg/d at 4–12 weeks before last menstrual period	Achieved optimal RBC folate levels (≥906 nmol/L)	[145]
ICR mice with diabetes-induced NTD	3 mg/kg/d with n-3 PUFA diet until sacrifice	Hcy ↓; NTD incidence ↓; apoptosis (apoptotic cells ↓; p53 ↓; Bax ↓); Pax3 methylation ↓; one-carbon metabolism (MTHFR ↑; MTR ↓; MAT ↓; Dnmt3b ↓; SAHH ↓; CBS ↑)	[151]
ICR mice with diabetic pregnancy	10 mg/kg for 7/8/10 d	NTD incidence ↓; Pax3 ↓; oxidative stress (ROS production ↓; MDA ↓; H_2_O_2_ ↓)	[148]
Wistar rats with cyclophosphamide-induced NTD	0.7 mg/kg (combined with 160 mg/kg of soy isoflavone) for 20 d	NTD incidence ↓	[129]
ICR mice with LPS-induced NTD	3 mg/kg/d from GD 8 to GD 12	NTD incidence ↓; inflammation (p-JNK ↑; p-IkB ↓; NF-κB p65 ↓; TNF-α ↓; IL-1β ↓; IL-6 ↓); oxidative stress (GSH ↓)	[147]
CD-1 mice with valproic acid-induced NTD	3 × 4 mg/kg/d from GD 5 to GD 10	Exencephaly ↓; apoptosis (p53 ↓; NF-κB ↑; Pim-1 ↑; c-Myb ↑; Bax/Bcl2 ↓)	[149]

Abbreviations: ↑, increase; ↓, decrease; AA, arachidonic acid; ADMA, asymmetric dimethylarginine; AKT, protein kinase B; AMPK, AMP-activated protein kinase; ANF, atrial natriuretic factor; Ang, angiotensin; ApoE^−/−^, apolipoprotein E-deficient; APP, amyloid precursor protein; Aβ, amyloid β; BA, butyric acid; BAK, Bcl-2 antagonist killer; Bax, Bcl-2 associated X-protein; Bcl-xL, B-cell lymphoma-extra large; Bcl-2, B-cell lymphoma-2; BDL, bile duct ligation; BNP, brain natriuretic peptide; BP, blood pressure; CBS, cystathionine-β-synthase; CD, cyclodextrin; Col, collagen; COX-2, cyclooxygenase-2; cPLA-2, cytosolic phospholipase-2; CRC, colorectal cancer; C/EBPα, CCAAT/enhancer-binding protein α; DFE, dietary folate equivalent; DHA, docosahexaenoic acid; DNMT1, DNA methyltransferase 1; DSS, dextran sulphate sodium; DTX, docetaxel; eNOS, endothelial NO synthase; EGF, epidermal growth factor; EPA, eicosapentaenoic acid; FA, folic acid; FABP-4, fatty acid binding protein-4; FADD, fas-associated death domain; Foxp3, forkhead box protein p3; GD, gestational day; GFR, glomerular filtration rate; GSH, reduced glutathione; GPx, glutathione peroxidase; GSSG, oxidized glutathione; Hcy, homocysteine; HSP-70, heat shock protein-70; H/L, heterophil/lymphocyte; IBA, isobutyric acid; ICR, Institute of Cancer Research; IFN-γ, interferon-γ; IGF-1, insulin-like growth factor-1; IL-1β, interleukin-1β; IVA, isovaleric acid; IκB, inhibitor of κB; JAK-STAT, Janus kinase-signal transducer and activator of transcription; JNK, c-Jun N-terminal kinase; LDL, low-density lipoprotein; LPS, lipopolysaccharide; LTD, long-term depression; MAT, methionine adenosyl transferase; MCP-1, monocyte chemoattractant protein-1; MDA, malondialdehyde; MMP, mitochondrial membrane potential; MMSE, mini-mental state examination; MTHFR, methylene THF reductase; MTR, 5-MTHF-Hcy methyltransferase; MTRR, methionine synthase reductase; NDV, Newcastle disease virus; NF-κB, nuclear factor-κB; NFAT, nuclear factor of activated T cell; NO, nitric oxide; NOX, nicotinamide adenine dinucleotide phosphate oxidase; NP, nanoparticle; NTD, neural tube defect; OPL, esophageal precancerous lesion; OPN, osteopontin; OSCC, esophageal squamous cell carcinoma; PCNA, proliferating cell nuclear antigen; PEG, polyethylene glycol; PGE2, prostaglandin-2; PLGA, poly lactic-co-glycolic acid; PLP, PEGylation liposome; pIL-12, plasmid IL-12; PI3K, phosphoinositide 3-kinase; PS, presenilin; PTEN, phosphatase and tensin homologue; PUFA, polyunsaturated fatty acid; RBC, red blood cell; RORγt; retinoid-related orphan nuclear receptor-γt; ROS, reactive oxygen species; SAHH, *S*-adenosylhomocysteine hydrolase; SAM, *S*-adenosylmethionine; SCFA, short-chain fatty acid; SHR, spontaneously hypertensive rat; SOD, superoxide dismutase; TAC, total antioxidant capacity; TBARS, thiobarbituric acid reactive substances; TC, total cholesterol; TFA, trans-ferulic acid; TG, triacylglycerol; TGF-β, transforming growth factor-β; THF, tetrahydrofolate; TNF-α, tumor necrosis factor-α; UACR, urinary albumin–creatine ratio; VEGF, vascular endothelial growth factor; VLDL, very-low-density lipoprotein; VSMC, vascular smooth muscle cell; α-SMA, α-smooth muscle act.

**Table 2:** Recommended dietary folate equivalents from the Academy of Nutrition and Dietetics.

Life Stage	Age	Males (µg/day)	Females (µg/day)
Infants	0–6 months	65	65
Infants	7–12 months	80	80
Children	1–3 years	150	150
Children	4–8 years	200	200
Children	9–13 years	300	300
Adolescents	14–18 years	400	400
Adults	>18 years	400	400
Pregnancy	-	-	600
Lactation	-	-	500

Adapted from [157].

## 6. Conclusions

Folate is an indispensable B group vitamin that can be found in a wide range of foods, including green leafy vegetables, fruits, legumes, and livers, with varying degrees of bioaccessibility and bioavailability. Applying various processing methods to food can release entrapped folate from the food matrix and make it bioaccessible, but this may compromise its stability. In this regard, encapsulation technology could be a viable way to keep folate intact during processing and storage and to help increase the bioavailability of folate in the body, thereby ensuring that people consume adequate levels of folate in their diets. Future research is encouraged to explore other innovative processing techniques that can enhance the bioaccessibility and bioavailability of natural folate while minimizing its degradation and liberation.

To combat folate deficiency, aside from directly fortifying crops with synthetic FA, folate can also be enriched by germination, fermentation, and genetic biofortification. Germination and fermentation are natural and sustainable ways to increase the folate content, but their yields are inconsistent and have the potential for pathogenic growth. On the other hand, metabolic engineering is a more reliable and efficient approach, but its acceptance by consumers and regulatory authorities may be a challenge. Greater emphasis should be placed on gene editing technologies, particularly CRISPR-Cas9, due to their proven ability to enhance certain nutrition values and their potential feasibility for folate enrichment.

Despite the several potential adverse effects associated with synthetic FA, it is still essential to consume it to fulfil the daily folate requirements when the intake of natural folate is inadequate. Numerous in vitro and in vivo studies have revealed many health benefits of folate and FA supplementation, including cardiovascular protection, hematopoiesis protection, neural tube protection, anti-dementia, anti-cancer, anti-hypertensive, and immune response enhancement, mainly through the regulation of Hcy levels, oxidative stress, and inflammation. In addition, folate could alter the gut microbiota composition, thereby contributing to the maintenance of gut homeostasis and the improvement of digestive health. Folate has also been found to mitigate depression by increasing the concentrations of monoamine neurotransmitters, while the connection between folate and the gut microbiota–brain axis has not been fully elucidated. Additional studies involving human subjects are warranted to establish the optimal dose and duration of folate supplementation for different health conditions and populations. Furthermore, the interactions of folate with other nutrients or medications should also be investigated to maximize its potential benefits.

## Supplementary Materials

The following supporting information can be downloaded at https://www.mdpi.com/article/10.3390/ijms26167703/s1.
