# Supplementary material for: A Comprehensive Review of Fortification, Bioavailability, and Health Benefits of Folate
Source: Int J Mol Sci. 2025 Aug 9;26(16):7703. doi: 10.3390/ijms26167703 (PMC12386915; doi:10.3390/ijms26167703)
Supplement: Supplementary file 1 [file ijms-26-07703-s001.zip › ijms-3796472-supplementary.pdf]

Table S1. Folate content in several common food items.

Food	Folate content (μg/100 g)
Livers	
Chicken, all classes, raw	588
Goose, raw	738
Duck, domesticated, raw	738
Beef, variety meats and by-products, raw	290
Pork, fresh, variety meats and by-products, raw	212
Legumes and pulses	
Beans, black, mature seeds, raw	444
Chickpeas, mature seeds, raw	557
Lentils, raw	479
Peanuts, all types, raw	240
Soybeans, mature seeds, raw	375
Fruits	
Avocados, raw, all commercial varieties	81
Bananas, raw	20
Oranges, raw, all commercial varieties	30
Pears, raw	7
Green leafy vegetables	
Spinach, raw	194
Turnip greens, raw	194
Lettuce, cos or romaine, raw	136
Kale, raw	62
Eggs	
Egg, whole, raw, fresh	47
Mushrooms	
Lentinus edodes, fresh, raw	25
Pleurotus ostreatus, fresh, raw	51
Oyster, fresh, raw	40
Commercial microalgae powders	
Chlorella powders	13.9 to 25.9
Spirulina powders	25 to 47
N. gaditana powders	20.8

Note: All data were obtained from the USDA website.
